# Construction of Oxygen-Rich Carbon Foams for Rapid Carbon Dioxide Capture

**DOI:** 10.3390/ma14010173

**Published:** 2020-12-31

**Authors:** Cheng Duan, Wei Zou, Zhongjie Du, Jianguo Mi, Jiaxi Han, Chen Zhang

**Affiliations:** 1 Key Laboratory of Carbon Fiber and Functional Polymers, Ministry of Education, College of Materials Science and Engineering, Beijing University of Chemical Technology, Beijing 100029, China; Beijing 100029, China; m18401587464@163.com (C.D.); zouwei@mail.buct.edu.cn (W.Z.); duzj@mail.buct.edu.cn (Z.D.); 2 State Key Laboratory of Organic-Inorganic Composites, Beijing University of Chemical Technology, mijg@mail.buct.edu.cn; 3 Huanqiu Contracting & Engineering Co., Ltd., Huanqiu Corporation, Beijing 100029, China; Hanjiaxi-hqc@cnpc.com.cn

**Keywords:** carbon foam, concentrated emulsion, specific surface areas, CO_2_ capture

## Abstract

As carbon dioxide (CO_2_) adsorbents, porous materials with high specific surface areas and abundant CO_2_-philic groups always exhibit high CO_2_ capacities. Based on this consensus, a category of oxygen-rich macroporous carbon foams was fabricated from macroporous resorcinol-formaldehyde resins (PRFs), which were obtained via an oil-in-water concentrated emulsion. By the active effect of potassium hydroxide (KOH) at high temperatures, the resultant carbon foams (ACRFs) possessed abundant micropores with rich oxygen content simultaneously. At the same time, most of the ACRFs could retain the marcoporous structure of their precursor. It is found that porosity of ACRFs was mainly determined by carbonization temperature, and the highest specific surface areas and total pore volume of ACRFs could reach 2046 m^2^/g and 0.900 cm^3^/g, respectively. At 273 K, ACRFs showed highest CO_2_ capacity as 271 mg/g at 1 bar and 91.5 mg at 15 kPa. Furthermore, it is shown that the ultra-micropore volume was mainly responsible for the CO_2_ capacities of ACRFs at 1 bar, and CO_2_ capacities at 15 kPa were mainly affected by the oxygen content. It is also found that the presence of macropores would accelerate ACRFs adsorbing CO_2_. This study provides ideas for designing a porous CO_2_ adsorbent.

## 1. Introduction

Being a greenhouse gas, the increasing content of carbon dioxide (CO_2_) in the atmosphere has been considered to be a key reason for the change of global climate, and thus CO_2_ capture technology has been developed [1]. Many works reported the investigation of porous materials as a CO_2_ adsorbent, including zeolite [2], metal-organic frameworks (MOFs) [3], porous organic polymers [4,5,6], and porous carbons [7], etc. Because of the excellent CO_2_ capacities and the exceptional chemical and physical stability, porous carbons have attracted much attention. It was already confirmed that the high CO_2_ capacities of porous carbon were related to the abundant micropore volumes and the heteroatom. For example, the carbons with a micropore volume of 0.93 and 1.05 cm^3^/g exhibited CO_2_ capacities of 3.3 and 2.1 mmol/g at 298 K, 1 bar [8,9]; N-doped porous carbons perform well in both CO_2_ capacities (4.3 and 5.2 mmol/g at 298 K, 1 bar) and selectivity (71 at 298 K, 1 bar, and 67 at 273 K, 1 bar) [10,11].

Besides CO_2_ uptake, the energy consumption of adsorbent also need to be considered in the practical industrial application, so macropores or mesopores were introduced into the adsorbent to minimize diffusion pressure in the adsorbent [12,13,14]. Therefore, it could be known that the hierarchical porous carbon would achieve an excellent comprehensive CO_2_ adsorption performance. Sacrifice templates, such as silicon monolith [15] and polystyrene nanoparticles [16], were applied for generating macropores in porous carbons. However, the removal of those template is a complicated and time-consuming process. Recently, the work on the fabrication of porous materials through concentrated emulsion template for the application of catalyst, separation, and adsorption has been reported [17,18,19]. Typically, the macroporous structures in polymers are generated through the removal of the internal phase, and generally an open-cell interconnected porous polymer monolith was obtained because of the droplet coalescence or Ostwald ripening during polymerization in the continuous phase.

**Scheme 1:**
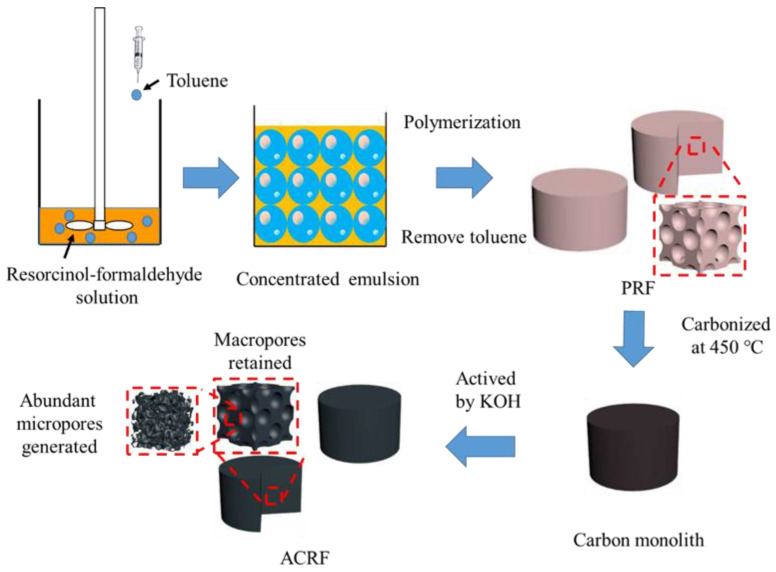
Preparation route of microporous-macroporous carbon foams (ACRFs).

In this work, a concentrated emulsion was applied for preparing a microporous-macroporous carbon foams (called as “ACRFs” in this paper), as illustrated in Scheme 1. Firstly, macroporous resorcinol-formaldehyde resins (PRFs) were fabricated via a concentrated emulsion, and then ACRFs were obtained by carbonizing PRFs with potassium hydroxide (KOH) as an activator. Attributed to the controlled carbonization process, the resultant ACRFs possessed high specific surface areas (over 2000 m^2^/g) with hierarchical porous structure, and also abundant oxygen-contained species on the surface. As a result, ACRFs exhibited as high CO_2_ capacity as 271 mg at 273 k and 1 bar. Furthermore, the effect of porosity, oxygen content and the presence of macropores on CO_2_ adsorbing performance of ACRFs were investigated. The results would be helpful for designing a carbon-based CO_2_ adsorbent.

## 2. Materials and Methods

### 2.1. Reagent

Formaldehyde (37 wt% aqueous solution, Xilong Scientific Co., Ltd., China), Resorcinol and Tween 20 (Tianjin Fuchen Chemical Reagent Factory, China), Anhydrous sodium carbonate and KOH (Tianjin Guangfu Fine Chemical Research Institute, China), Toluene, methanol, and ethanol (Beijing Chemical Works, China). All reagents were used as received.

### 2.2. Preparation of PRF

Resorcinol (2.20 g), formaldehyde aqueous solution (3.24 g), anhydrous sodium carbonate (0.04 g) and deionized water (1.36, 3.06, or 5.89 g) were added into a 100 mL flask and pre-polymerized at 40 °C for 30 min, respectively. Then, the resorcinol-formaldehyde solution with the 50 wt%, 40 wt%, or 30 wt% concentration was obtained after cooling down to room temperature. The resultant solution (5 mL), which acted as the continuous phase of the concentrated emulsion, was mixed with Tween 20 (0.86 g) in 100 mL round-bottom flask with a D-shape stirring paddle on the top. Then, toluene was added slowly under continuous stirring as the dispersed phase. Finally, a light-pink emulsion was formed. After the emulsion was stirred homogeneously, it was transferred into a mould and solidified in an oven at 60 °C. During this process, PRFs were obtained and the toluene droplets would leave unique open-cell macropores simultaneously. The usage of toluene was 20, 28.5, or 45 ml for the emulsion containing 80 vol%, 85 vol%, or 90 vol% of the dispersed phase, respectively. The formula of the PRFs was listed in Table S1.

### 2.3. Preparation of ACRF

In a typical carbonizing procedure, PRF monolith (the sizes of monoliths were prepared as 60 mm^3^ approximately) was placed in a tube furnace (CHY-1200, Henan Chengyi Instrument Technology Co. Ltd., Zhengzhou, China), and heated to 450 °C with a ramp rate of 10 °C/min. Subsequently, the pre-carbonized PRF was soaked in water/methanol solution with dissolved KOH (the mass ratio of KOH to PRFs was 2:1) for 3 h. After the solution was removed by rotary evaporation, the precursor impregnated by KOH was obtained. Thirdly, the precursor was heated to 600, 700, or 800 °C with a ramp rate of 10 °C/min below 450 °C and 5 °C/min above 450 °C. After carbonizing for 1 h, ACRF was formed. The ACRF was cooled down to room temperature, washed by deionized water for several times to remove the KOH, and then dried in a vacuum oven. The formula of the ACRFs was listed in Table S1.

### 2.4. Characterization

Fourier transform infrared spectroscopy (FT-IR) spectra were measured by a Nicolet-Nexus 670 Spectrometer (ThermoFisher Scientific, Waltham, MA, USA). X-ray photoelectron spectroscopy spectra (XPS) were obtained by ESCALAB 250 (ThermoFisher Scientific, USA) with a monochromated Al Kalph X-ray source. Macropores in PRFs and ACRFs were observed by scanning electron microscopy (SEM, S-4700, JEOL Ltd., Japan). N_2_ adsorption–desorption isotherms were measured by a JW-BK122W gas sorption analyzer (JWGB Sci. & TECH, Beijing, China) at 77 K. Specific surface areas of the samples were calculated according to Brunauer–Emmett–Teller (BET) theory, and the pore size distributions were determined according to DFT model with slit pore geometry. CO_2_ adsorption isotherms were also measured by the JW-BK122W gas sorption analyzer. Raman spectra were obtained by a Renishaw inVia micro-Raman spectrometer (Renishaw, London, UK), with 532 nm (2.33 eV) wavelength laser focused through an inverted microscope. X-ray diffraction (XRD) patterns were acquired on an Ultima IV diffractmeter (Rigaku, Tokyo, Japan) in the scanning range (2θ) of 10 to 90°.

CO_2_ adsorption kinetics was examined by TA Q500 thermo gravimetric analyzer (TA Instrument, Newcastle, CA, USA). Sample (6.5 mg) was placed in the ceramic pan, then it was heated to 300 °C with the rate of 15 °C/min under N_2_ flow (40 mL/min) and kept for 1 h to remove pre-adsorbed gases and moisture. Afterwards, the temperature was decreased to 40 °C, and the gas was switched to CO_2_ with the flow rate of 40 ml/min. The CO_2_ adsorbing speed was obtained by recording the weight change of the sample with time.

The breakthrough experiment was carried out in BSD-MAB (BEISHIDE instrument, Beijing, China) at 298 K. The sample (0.76 g) in a test tube with the internal diameter of 10 mm was pretreated at 300 °C for 2 h before test. Subsequently, CO_2_ (2 sccm), N_2_ (11.33 sccm, CO_2_/N_2_ was 15/85) and helium (50 sccm, as carrier gas) were mixed and used for column breakthrough curve measurement.

## 3. Results and Discussion

### 3.1. Carbonizing Process of ACRFs

**Figure 1:**
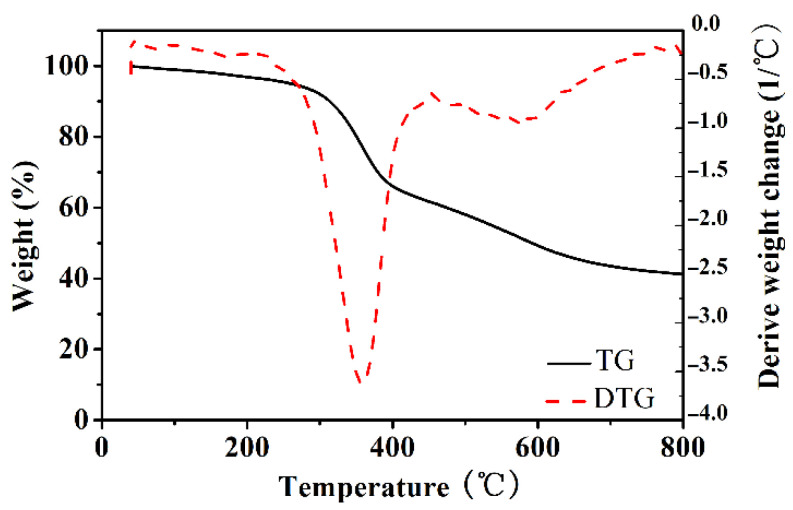
TGA and DTG curves of PRF-50C-85% under nitrogen flow.

It was well known that using KOH as an activator during the carbonization process could generate abundant micropores in the resultant carbon [20,21,22]. Impregnating KOH aqueous solution was an effective way for loading KOH into the macroporous precursor without damaging macropores. However, it was difficult to load KOH solution in PRFs is because of hydrolysis. To solve this problem, PRFs were firstly carbonized at low temperature, then the resultant macroporous carbon could be impregnated with KOH aqueous solution and further activated at the demanded temperature. TGA/DTA curves were used for determining the pre-carbonizing temperature and activation temperature (Figure 1). The weight loss in 100–220 °C (about 5 wt%) should be attributed to the loss of moisture and residue small molecules. The primarily weight loss of 34.1 wt% was in 250–450 °C, which was due to the decomposition of the phenolic group and ether group. Finally, the weight loss was continued in 450–800 °C with the carbonization process following. Accordingly, the carbonization process was set as follows—the pre-carbonized temperature was set at 450 °C to turn organic PRFs into chemical-stable carbon, and the obtained carbon was further activated with KOH at 600, 700 and 800 °C, respectively. (XRD patterns and Raman spectra of ACRFs prepared at 600, 700 and 800 °C were presents in Figure S1).

### 3.2. Oxygen in Porous ACRFs

**Figure 2:**
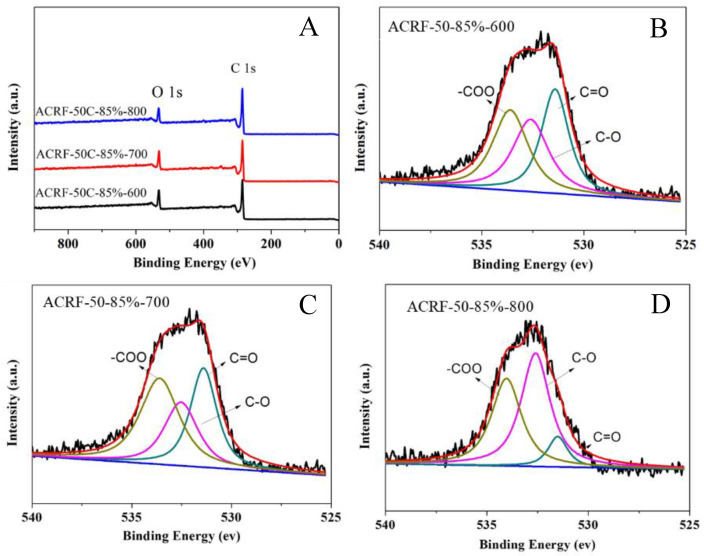
X-ray photoelectron spectroscopy (XPS) spectra of all ACRFs (**A**) and deconvolution of O 1s peaks of ACRF-50-85%-600 (**B**), ACRF-50-85%-700 (**C**) and ACRF-50-85%-800 (**D**).

It is of great interest to investigate the influence of carbonization temperature on the chemical structure of ACRFs, especially the oxygen-containing species in ACRFs. XPS spectra of ACRF-50C-85%-600, ACRF-50C-85%-700, and ACRF-50C-85%-800 were applied for investigating the surface chemistry state of these carbons (Figure 2). The characteristic peaks at 284.8 and 531.8–532.8 eV presented C 1s and O 1s, respectively, which illustrated that there was oxygen doped in the ACRFs. The deconvolution of O 1s peaks indicated the existence of carbonyl or quinone groups (531.5 eV), ether linkage or alcohol (532.5 eV), and a carboxyl group (533.5 eV) [23]. According to the XPS spectra, the oxygen in ACRF-50C-85%-600, ACRF-50C-85%-700, and ACRF-50C-85%-800 was about 18.7 wt%, 18.0 wt%, and 14.6 wt%, respectively, which indicated that ACRFs were oxygen-enriched. (The oxygen element content at different chemical state in different ACRFs was presented in Table S2) These data also showed that increasing the carbonization temperature from 600–700 °C would not change the oxygen content or chemical state considerably. However, the oxygen content in ACRFs would reduce rapidly with the increase of carbonization temperature when it is over 700 °C. It is well known that KOH could react with carbon as follows [24]:

6KOH + 2C → 2K_2_CO_3_ + 3H_2_ + 2K (1)

When the temperature is over 700 °C:

K_2_CO_3_ → K_2_O + CO_2_ (2)

K_2_CO_3_ + 2C → 2K + 3CO (3)

K_2_O+C → 2K + CO (4)

CO_2_ + C → 2CO (5)

These reactions showed that the oxidization of the carbon atom would be enhanced when the temperature was over 700 °C, which meant more oxygen atom in the carbon would be consumed during the carbonization process. That was the reason for lower oxygen content in ACRF-50C-85%-800 than ACRF-50C-85%-600 or ACRF-50C-85%-700. 

### 3.3. Porosity of ACRFs

**Figure 3:**
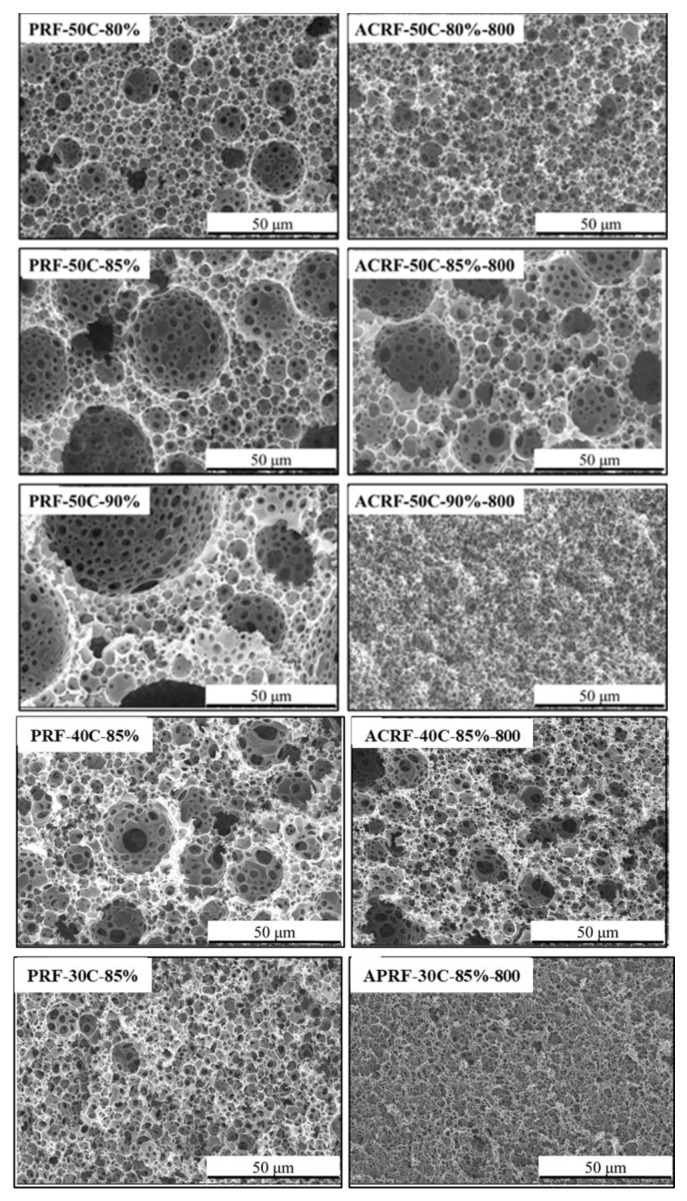
Scanning electron microscope (SEM) images of PRFs and corresponding ACRFs.

The macroporous morphologies of PRFs and ACRFs were presented in Figure 3. For all the PRFs, the typical SEM images of the porous material obtained via concentrated emulsion template could be seen [25,26]—there were many closely-packed cavities (which were called “voids”) interconnected with the adjacent ones by small pores (which were called “windows”). It was found that the size of voids could be tuned by adjusting the composition of concentrated emulsion—in PRF-50C-80%, PRF-50C-85%, and PRF-50C-90% the size of voids increased with the increasing dispersed phase volume fraction in concentrated emulsion, and in PRF-50C-85%, PRF-40C-85%, and PRF-30C-85% the sizes of voids decreased with the decreasing concentration of continuous phase in concentrated emulsion. For ACRFs, ACRF-50C-80%-800, ACRF-50C-85%-800, and ACRF-40C-85%-800 all inherited the marocporous structures of their corresponding PRFs. However, the size of macropores in ACRF-50C-90%-800 and ACRF-30C-85%-800 shrank obviously, which illustrated that the macropores in PRF-50C-90% and PRF-30C-85% collapsed during the carbonization process [27]. The marcoporous morphologies of ACRF-50C-85%-600, ACRF-50C-85%-700, and ACRF-50C-85%-800 were similar (Figure S2), indicating that the influence of carbonization temperature on macropores in ACRFs was limited. In general, the macroporous structure of ACRFs could be tuned by concentrated emulsion to some extent.

**Figure 4:**
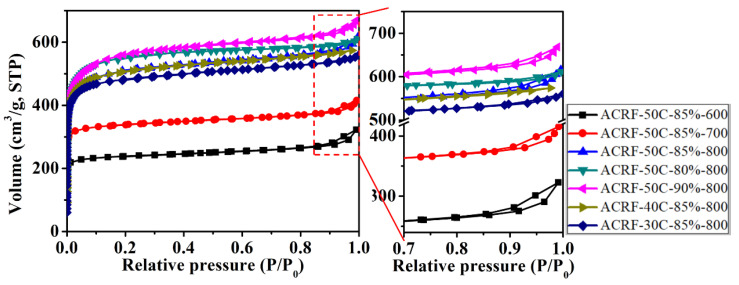
N_2_ adsorption-desorption isotherms of ACRFs at 77 K.

**Figure 5:**
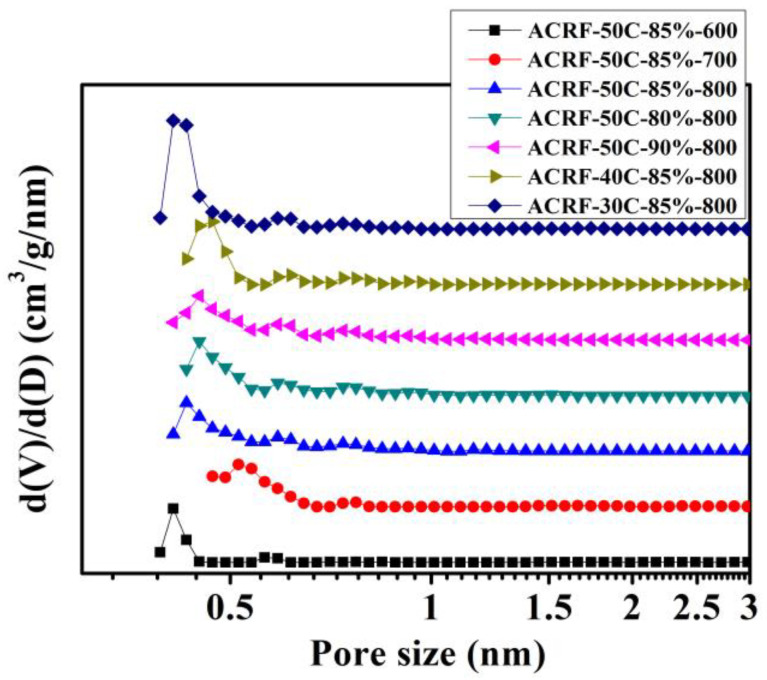
Pore size distributions of ACRFs.

N_2_ adsorption-desorption isothermals of all the ACRFs were exhibited in Figure 4. (Isothermals at low at low relative pressure were presented in Figure S3) All isothermals showed steep gas uptake at relatively low pressure (P/P_0_ < 0.01), indicated there were abundant micropores in ACRFs [28], and the slight increase of adsorption at relatively high pressure (P/P_0_ > 0.9) indicated the existence of macropores [29]. It could be summarized that increasing carbonization temperature could increase the specific surface areas (S_BET_) and pore volumes of ACRFs greatly—with the increase of carbonization temperature from 600 °C to 800 °C, the S_BET_, total pore volumes (V_tot_) and micropore volumes (V_mic_) of ACRFs greatly increased from 879 to 1952 m^2^/g, 0.433 to 0.830 cm^3^/g, and 0.349 to 0.742 cm^3^/g, respectively. The increase of porosity should be attributed to the etching effect on carbon atoms by oxidation, in situ generated potassium intercalating within carbon layers, and the generated CO_2_ reacting with carbon via the carbon gasification process to further promote the development of porosity [24]. It is also confirmed that the influence of dispersed phase volume ratio or concentration of continuous phase in concentrated emulsion on the textural properties was limited. The pores in all of the ACRFs were dominantly micropores—the volume of micropores possessed 80.6–96.3% of the total pore volumes. To be more specific, most of the micropores in ACRFs were ultra-micropores (sizes below 0.7 nm)—pore size distributed mainly at 0.41 nm in ACRF-50C-85%-600 and ACRF-30C-85%-800; in ACRF-50C-85%-700, the dominant size of pores was 0.52 nm; in other ACRFs, most of the pores were around 0.44 nm. (Figure 5).

### 3.4. CO_2_ Capacity of ACRFs at 1 Bar

**Figure 6:**
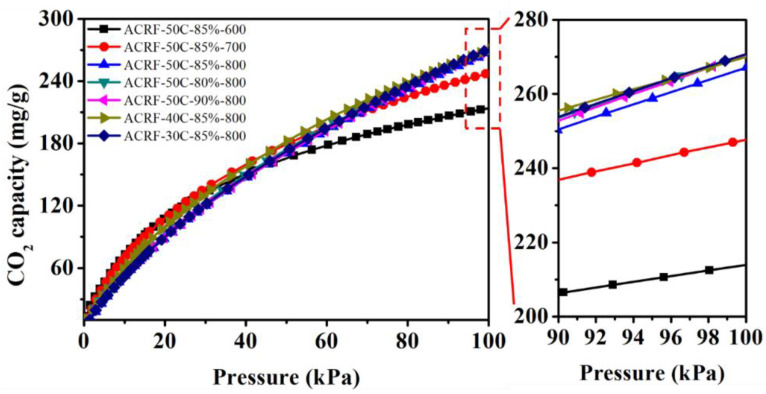
CO_2_ capacities of ACRFs at 273 K.

**Figure 7:**
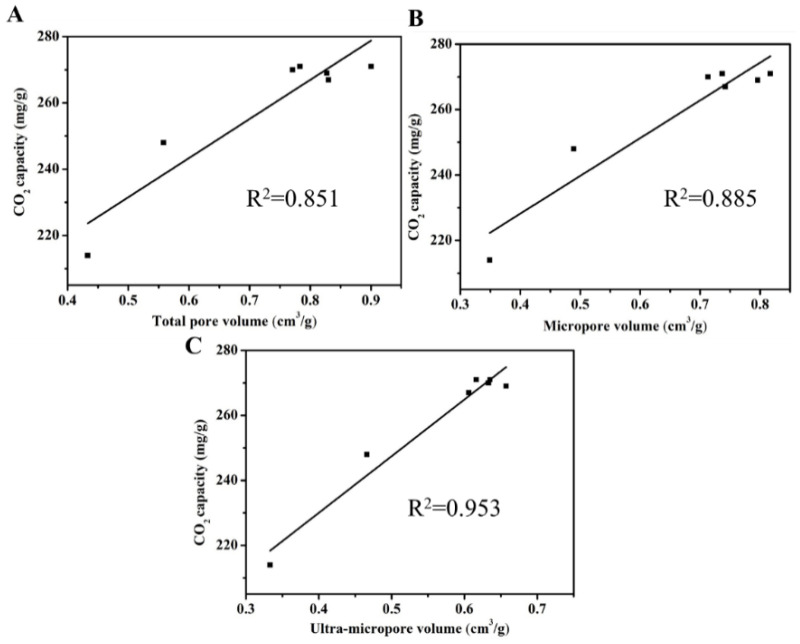
Relationships between CO_2_ capacities and total pore volume (**A**), micropore volume (**B**), and ultra-micropore volume (**C**).

It could be expected that ACRFs could exhibit high CO_2_ uptake because of the abundant micropores [30,31,32]. The CO_2_ adsorption isothermals of all the ACRFs were presented in Figure 6 and summarized in Table 1. Except for ACRF-50C-85%-600 and ACRF-50C-85%-700, ACRFs showed CO_2_ capacities around 267–271 mg/g, which were comparable to many reported carbon materials (Table S3). In order to further investigate the factor that would determine CO_2_ capacity of ACRFs at 1 bar, relationships of CO_2_ capacities with porosity were presented in Figure 7. It was shown that the linear correlation coefficients (R^2^) of V_tot_ versus CO_2_ capacity, or V_micr_ versus CO_2_ capacity were below 0.9; by contrast, V_utra_ showed high linear correlation with CO_2_ capacity, which implied that ultra-micropores in ACRFs were essential for CO_2_ capacities at 1 bar. The high adsorption potential of ultra-micropores would be the factor that would be the determining factor of CO_2_ capacity at 1 bar [15].

### 3.5. Effect of Oxygen Content on the CO_2_ Capacities of ACRFs

Here ACRF-50C-85%-600, ACRF-50C-85%-700, and ACRF-50C-85%-800 were used for investigating the influence of oxygen content on the CO_2_ capacity of ACRFs. It is interesting to find that ACRF-50C-85%-600 and ACRF-50C-85%-700 showed a CO_2_ capacity of 91.7 and 90.5 mg/g at 15 kPa, respectively, which was higher than ACRF-50C-85%-800 (71.6 mg/g). According to Section 3.2, it could be found that CO_2_ capacity of ACRFs at 15 kPa increased with increasing oxygen content. The oxygen on the surface was considered to enhance the affinity and interaction between adsorbent and CO_2_ [33], so it is believed that the increase of oxygen in ACRFs would enhance the interaction between carbon surface and the CO_2_ molecule, which is essential for ACRFs capturing CO_2_ at low pressure. It should be noted that flue gas always contains about 15% CO_2_ at total pressures of around 1 bar, so the CO_2_ capacity of adsorbent at 15 kPa is more relevant to realistic performance [1]. (Relationships between CO_2_ capacities at 15 kPa and the content of oxygen in different chemical state were presented in Figure S4).

### 3.6. Effect of Macropores on CO_2_ Adsorption Kinetics of ACRFs

**Figure 8:**
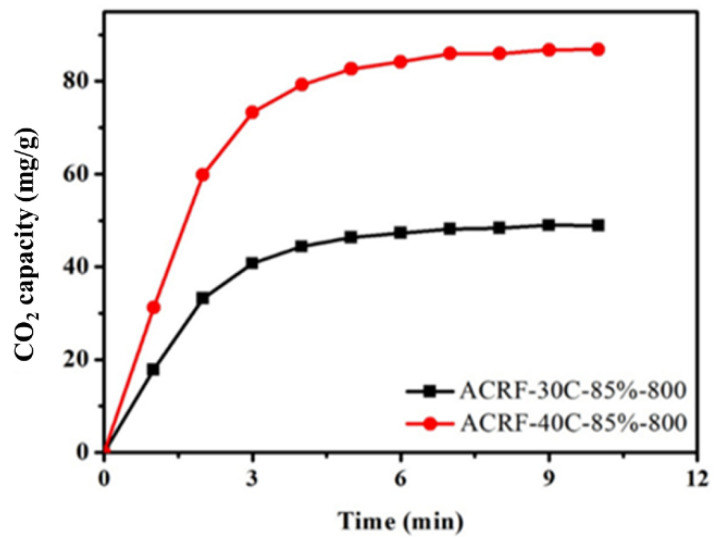
Comparison of the CO_2_ adsorption kinetics of ACRF-40C-85%-800 and ACRF-30C-85%-800.

Previous works considered that macropores would provide a high adsorption rate by reducing the diffusion distance and increasing mass transfer rate [34]. Here, we wanted to find some evidence for this view. In Figure 8, CO_2_ adsorption kinetics of ACRF-30C-85%-800 and ACRF-40C-85%-800 were presented. ACRF-40C-85%-800 exhibited faster adsorption kinetics than ACRF-30C-85%-800. This difference could not be attributed to the micropores or mesopores in ACRFs, because there was only little difference between ACRF-30C-85%-800 and ACRF-40C-85%-800 on S_BET_ or pore volume. So, it is believed that the slower adsorption rate of ACRF-30C-85%-800 should be due to its severely damaged macropores. In this way, the accelerating effect of macropores on CO_2_ adsorption kinetics was confirmed.

### 3.7. CO_2_ Adsorbing Properties of ACRF-40C-85%-800

**Figure 9:**
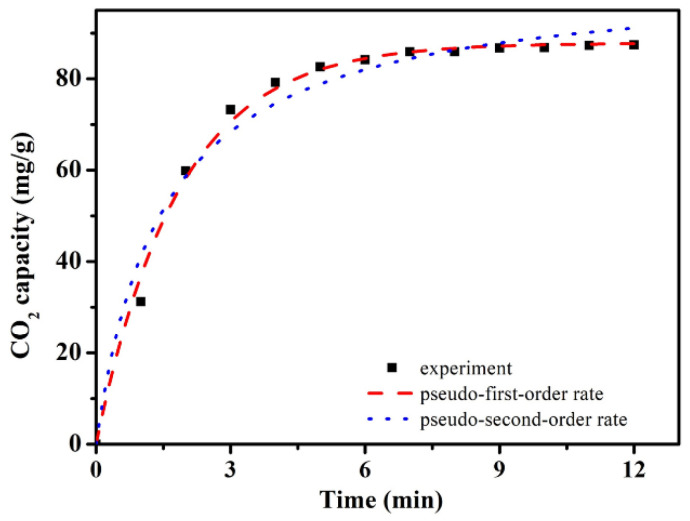
CO_2_ adsorption kinetics of ACRF-40C-85%-800.

As the typical carbon foam sample, further CO_2_ adsorbing behaviors of ACRF-40C-85%-800 were investigated. Firstly, the CO_2_ adsorption kinetics experimental data were fixed by a pseudo-first-order rate model (Equation (7)) and pseudo-second-order rate model (Equation (8)) in Figure 9.

(6) $$\ln\left(q_{e}{\;-\;q}_{t}\right){=-k}_{1}{t+\mathrm{lnq}}_{e}$$

(7) $$\frac{t}{q_{t}}=\frac{t}{q_{e}}\;+\;\frac{1}{k_{2}q_{e}^{2}}$$

where q_e_ and q_t_ are the CO_2_ adsorption capacity (mmol/g) at equilibrium and at time t (min), respectively; k_1_ is the adsorption rate constant of pseudo-first order (min^−1^), and k_2_ is the rate constant of pseudo-second order adsorption (g/mg·min). The corresponding parameters were summarized in Table 2. From the value of R^2^, it ensured that the pseudo-first-order rate model was more suitable for describing the adsorption behavior of ACRF-40C-85%-800 than the pseudo-second-order model, which indicated that the adsorption process was controlled by diffusion steps, and the interactions between carbon foam and CO_2_ is reversible at equilibrium [35].

The micropore diffusion model was also used to analyze the kinetics data [36,37]. Like the results of the pseudo-second-order rate model, there was obvious deviation between the fitting data from micropore diffusion model and experimental data (Figure S5 and Table S4, illustrating that the microporous diffusion model was less suitable for describing the adsorption behavior.

**Figure 10:**
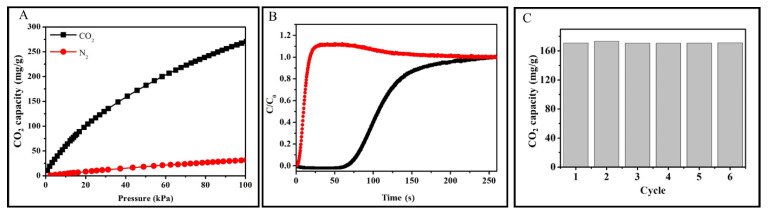
CO_2_ and N_2_ adsorption isothermals of ACRF-40C-85%-800 at 273 K (**A**); breakthrough curves of CO_2_/N_2_ of ACRF-40C-85-800 at 298 K (**B**); CO_2_ capacity at 298 K of ACRF-40C-85%-800 for six cycles (**C**).

Secondly, the performance of CO_2_ selectively adsorption of ACRF-40C-85%-800 was also measured. The CO_2_ selectivity adsorption over N_2_ was evaluated by the adsorption isotherms of CO_2_ and N_2_ at 273 K (Figure 10A). The calculated selectivity was 17.6 on the basis of the ideal adsorption solution theory (IAST) [38]. In order to better investigate the performance of ACRF-40C-85%-800 in practical applications, a breakthrough experiment was also performed (Figure 10B). It could be seen that the N_2_ broke through the test tube after only 3.7 s, and the concentrate of N_2_ at the outlet of the column was even higher than the concentrate of N_2_ in the initial gas (C/C_0_ > 1) in 250 s; on the contrary, it took 74 s for CO_2_ to break though the test tube, much longer than N_2_. These results indicated the competitive adsorption process—much N_2_ occupied the activity site of carbon at the initial stage because of its high concentration in mixed gas, but then the adsorbed N_2,_ molecules were replaced by CO_2_ molecules, and the desorbed N_2_ increased the concentrate of N_2_ in flow [39]. In a word, ACRF-40C-85%-800 tended to adsorb CO_2_ over N_2_, and it could selectively capture CO_2_ over N_2_ from a mixed gas feed stream.

Thirdly, the reproducibility of ACRF-40C-85%-800 was shown in Figure 10C. After six cycles, ACRF-40C-85%-800 showed the same capacity as the first cycle (171 mg/g), which illustrated that ACRF-40C-85%-800 had good reproducibility.

**Figure 11:**
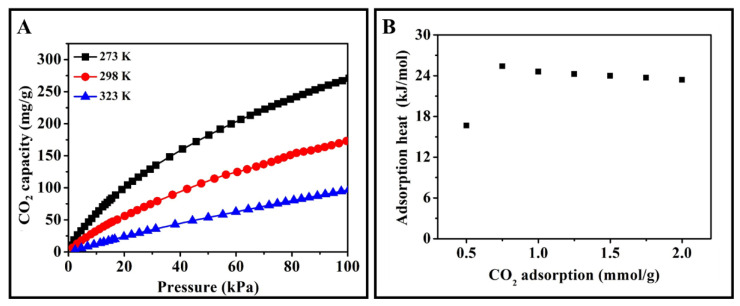
CO_2_ adsorption isothermals (**A**) and CO_2_ adsorption heats (**B**) of ACRF-40C-85%-800.

At last, the CO_2_ adsorption heat was calculated by Clausius−Clapeyron equation via the CO_2_ adsorption isothermals at 273, 298, and 323 K (Figure 11A) [40]:

(8) $${\left(\frac{\partial \mathrm{lnP}}{\partial \left(1/T\right)}\right)}_{\theta }=-\frac{Q_{\mathrm{st}}}{R}$$

where P is pressure of certain CO_2_ adsorbing capacity, T is the relative CO_2_ adsorbing temperature, θ is the amount of adsorbed CO_2_, and R is the universal gas constant. The CO_2_ adsorption heats were around 16.7–24.5 kJ/mol (Figure 7B). These moderate adsorption heats meant ACRF-40C-85%-800 could adsorb CO_2_ effectively, and the energy cost for regeneration would be acceptable.

## 4. Conclusions

A strategy of preparing monolith-like carbon (ACRFs) with macropores and micropores simultaneously by carbonizing the precursor obtained using concentrated emulsion template was reported. The micropores in the carbon were generated by the activation effect of KOH, and the macropores in the carbon were derived from the macroporous precursor. The S_BET_, V_tot_, and V_mic_ of this carbon were mainly controlled by carbonization temperature, and the macropores in this carbon could be tuned by the dispersed phase volume ratio in the emulsion. Among these carbons, ACRF-50C-85%-900 showed the highest specific surface area of 2046 m^2^/g, with total pore volume of0.900 cm^3^/g. As a CO_2_ adsorbent, it is found that the ultra-micorpore volume would determine the CO_2_ capacities of ACRFs at 1 bar, and the oxygen content would greatly affect the CO_2_ capacities of ACRFs at 15 kPa. It is also noticeable that the macropores in ACRFs would accelerate the CO_2_ adsorption rate. The highest CO_2_ capacities of this carbon were 271 mg/g at 273 K, 1 bar (ACRF-50C-85%-800 and ACRF-40C-85%-800), and 91.7 mg/g at 15 kPa (ACRF-50C-85%-600). ACRFs also exhibited moderate CO_2_ adsorption heat and selectively adsorption ability, so it is believed that ACRFs had the potential to be developed into a CO_2_ adsorbent with outstanding comprehensive performance.

## Figures and Tables

**Table 1:** Specific surface areas, pore volume and CO_2_ capacities of ACRFs.

Carbon Foam	Specific Surface Areas ^1^ m^2^/g	Total Pore Volume ^2^ cm^3^/g	Micropore Volume ^3^ cm^3^/g	Ultra–Micropore Volume ^3^ cm^3^/g	Micropore Volume/Total Pore Volume	CO_2_ Capacity ^4^ mg/g
ACRF-50C-85%-600	879	0.433	0.349	0.333	0.806	214
ACRF-50C-85%-700	1237	0.558	0.489	0.466	0.876	248
ACRF-50C-85%-800	1952	0.830	0.742	0.606	0.894	267
ACRF-50C-80%-800	2024	0.827	0.796	0.657	0.963	269
ACRF-50C-90%-800	2046	0.900	0.817	0.635	0.908	271
ACRF-40C-85%-800	1944	0.783	0.737	0.616	0.941	271
ACRF-30C-85%-800	1871	0.771	0.713	0.633	0.925	270

^1^ Calculated BET surface area over the pressure range of 0.01–0.2 P/P_0_ values. ^2^ Calculated by DFT method, cumulative pore volume under 2 nm. ^3^ Calculated by DFT method, cumulative pore volume under 0.7 nm. ^4^ Measured at 273 k, 1 bar.

**Table 2:** Kinetics model parameters for CO_2_ adsorption.

Sample	Pseudo-First-Order Model		Pseudo-Second-Order model	
ACRF-40C-85%-800	q_e_ (mg/g)	87.8	q_e_ (mg/g)	102
	k_1_ (min^−1^)	0.544	k_2_ (min^−1^)	0.00655
	R^2^	0.994	R^2^	0.975

## Data Availability

Data sharing is not applicable to this article.

## Supplementary Materials

The following are available online at https://www.mdpi.com/1996-1944/14/1/173/s1, Figure S1: XRD patterns (A) and Raman spectra (B) of ACRFs prepared at different carbonization temperatures, Figure S2: SEM images of carbon foams prepared at different carbonizing tempera-tures , Figure S3: N2 adsorption-desorption isotherms of ACRFs at 77 K at low relative pressure, Figure S4: Relationships between CO_2_ capacities (at 15 kPa) of ACRFs and the content of oxygen in different chemical state; Figure S5: CO_2_ adsorption kinetics of ACRF-40C-85%-800 fitted by mi-cropore diffusion model. Table S1: Formula of PRFs and ACRFs, Table S2: Oxygen element con-tent at different chemical state in the carbon foams obtained from curve fitting of the O 1s spectra, Table S3: Summary of BET specific surface area and CO_2_ capture performance comparison of various solid physical adsorbent, Table S4: CO_2_ adsorption kinetics of ACRF-40C-85%-800 fitted by micropore diffusion model.
