# Supplementary material for: Construction of Oxygen-Rich Carbon Foams for Rapid Carbon Dioxide Capture
Source: Materials (Basel). 2020 Dec 31;14(1):173. doi: 10.3390/ma14010173 (PMC7795872; doi:10.3390/ma14010173)
Supplement: Supplementary file 1 [file materials-14-00173-s001.pdf]

Supporting Information

Construction of Oxygen-Rich Carbon Foams for Rapid Carbon Dioxide Capture

Cheng Duan [1,2], Wei Zou [1], Zhongjie Du [1], Jianguo Mi [2], Jiaxi Han [3], and Chen Zhang [1,*]

1 Key Laboratory of Carbon Fiber and Functional Polymers, Ministry of Education, College of Materials Science and Engineering, Beijing University of Chemical Technology, Beijing 100029, China
2 State Key Laboratory of Organic-Inorganic Composites, Beijing University of Chemical Technology, Beijing 100029, China
3 Huanqiu Contracting & engineering Co., Ltd. Huanqiu Corporation, Beijing 100029, China
* Correspondence: zhangch@mail.buct.edu.cn

Table S1. Formula of PRFs and ACRFs.

Sample	Water Added in Continuous Phase	Concentration of Continuous Phase	Toluene	Volume of Dispersed Phase	Carbonized Temperature
PRF-50C-80%,	1.36 g	50 wt%	20 ml	80 vol%	-
PRF-50C-85%,	1.36 g	50 wt%	28.5 ml	85 vol%	-
PRF-50C-90%,	1.36 g	50 wt%	45 ml	90 vol%	-
PRF-40C-85%,	3.06 g	40 wt%	28.5 ml	85 vol%	-
PRF-30C-85%,	5.89 g	30 wt%	28.5 ml	85 vol%	-
ACRF-50C-80%-800	1.36 g	50 wt%	20 ml	80 vol%	800℃
ACRF-50C-85%-800	1.36 g	50 wt%	28.5 ml	85 vol%	800℃
ACRF-50C-90%-800	1.36 g	50 wt%	45 ml	90 vol%	800℃
ACRF-50C-85%-700	1.36 g	50 wt%	28.5 ml	85 vol%	700℃
ACRF-50C-85%-600	1.36 g	50 wt%	28.5 ml	85 vol%	600℃
ACRF-40C-85%-800	3.06 g	40 wt%	28.5 ml	85 vol%	800℃
ACRF-30C-85%-800	5.89 g	30 wt%	28.5 ml	85 vol%	800℃

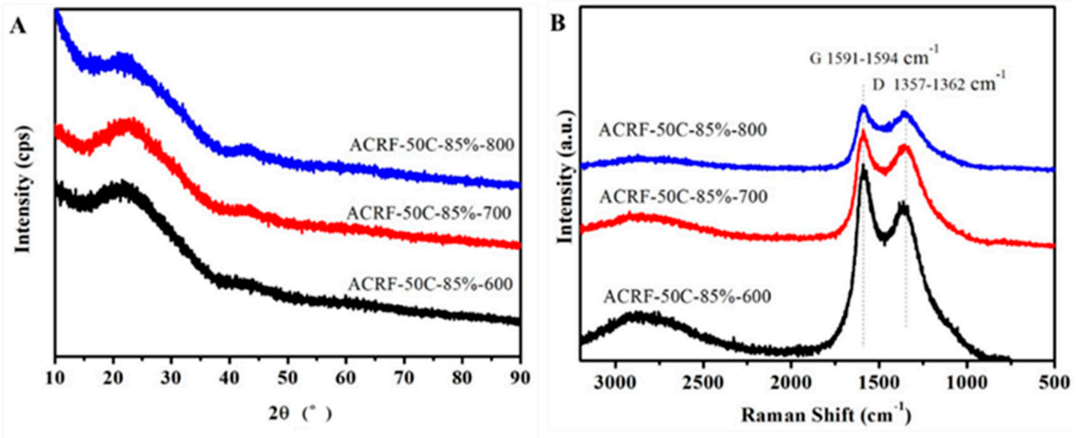

Figure S1. XRD patterns (A) and Raman spectra (B) of ACRFs prepared at different carbonization temperatures.

In Figure S1, the XRD patterns of ACRF-50C-85%-600, ACRF-50C-85%-700, and ACRF-50C-85%-800 showed similar diffraction patterns with board bands at 2θ = 23°and weak bands at 2θ = 43°, which was corresponded to (002) and (100) diffraction planes of graphitic carbon, respectively. It is revealed that the graphitization degree in these ACRFs were low [1,2]. Furthermore, the Raman spectra were measured and shown in Figure 6B. The G band (1591–1594 cm^{-1}) was associated with the stretch modes of sp^2-bonded C, which represented the graphitic domains in the materials, and

* Corresponding author, Email: zhangch@mail.buct.edu.cn

the D band (1357–1362 cm⁻¹) was attributed to the breathing mode of k-point phonons A_{1g} symmetry, which represented disordered and defective domains of graphite [3]. The intensity ratios of D band to G band were 0.86 (ACRF-50C-85%-600), 0.89 (ACRF-50C-85%-700), and 0.91 (ACRF-50C-85%-800), respectively. It could be known that the ratio of disordered and defective domains in carbon foam increased as the carbonized temperature increasing.

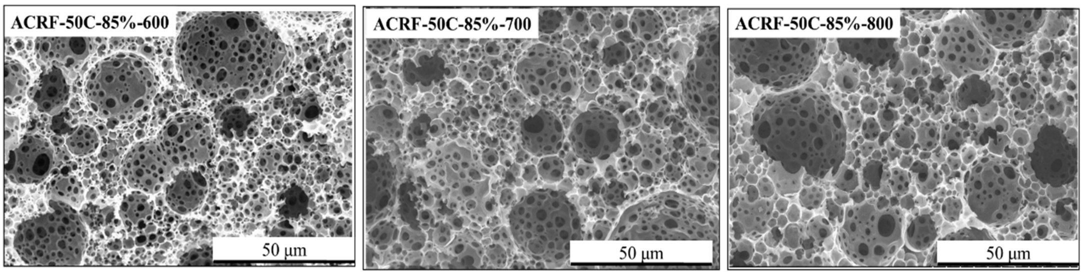

Figure S2. SEM images of carbon foams prepared at different carbonizing temperatures.

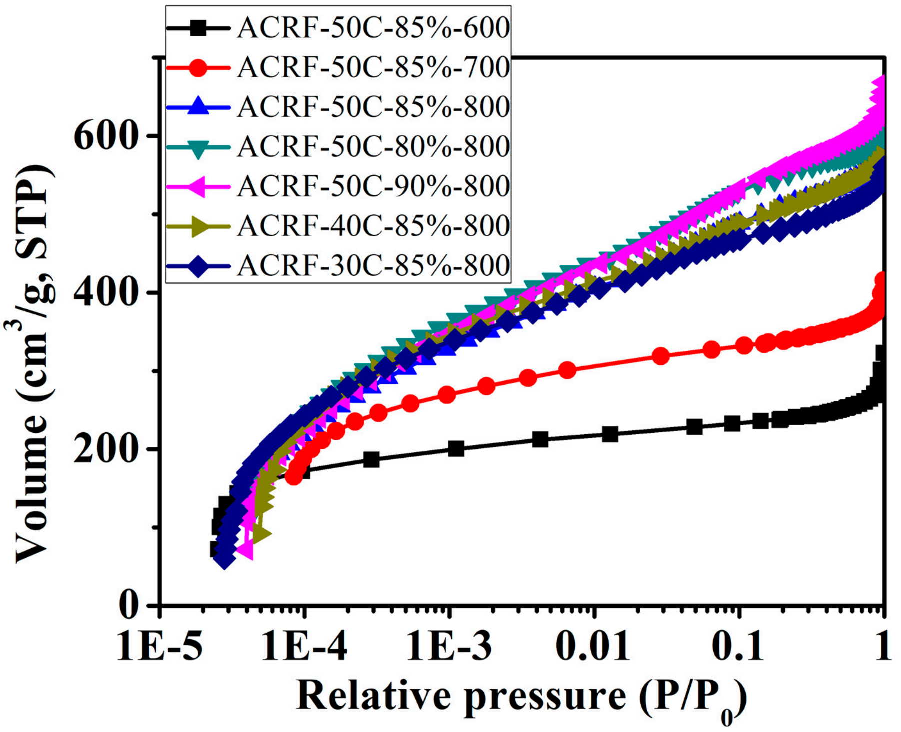

Figure S3. N_2 adsorption-desorption isotherms of ACRFs at 77 K at low relative pressure.

Table S2. Oxygen element content at different chemical state in the carbon foams obtained from curve fitting of the O 1s spectra.

Carbon Foam	Total O Content wt%	C=O Content wt%	C-O Content wt%	-COO Content wt%
ACRF-50C-85%-600	18.7	6.40	5.98	6.32
ACRF-50C-85%-700	18.0	6.41	5.09	6.50
ACRF-50C-85%-800	14.6	1.33	7.40	5.87

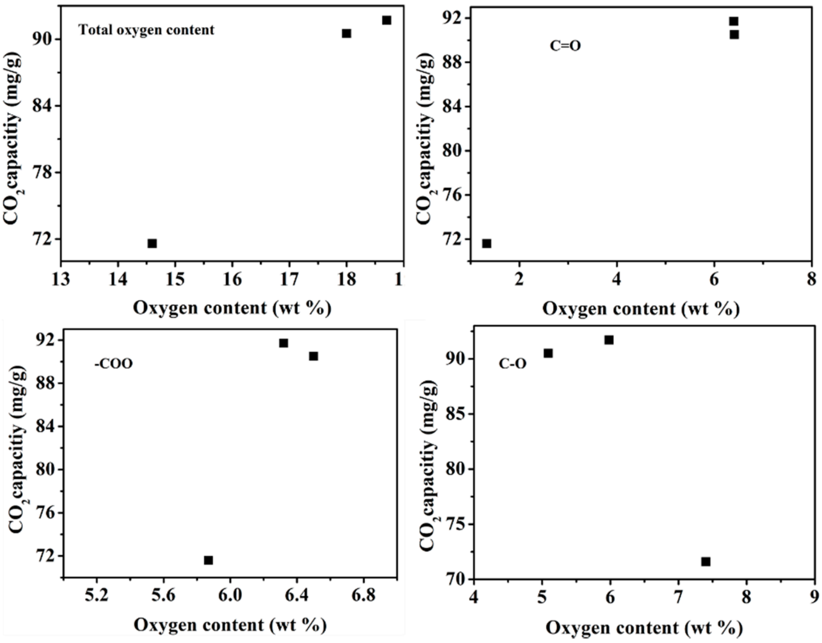

Figure S4. Relationships between CO_2 capacities (at 15 kPa) of ACRFs and the content of oxygen in different chemical state.

From Figure S4, it could be found that there was some relationship between CO_2 capacities at 15 kPa and total oxygen content, but it is hard to tell the effect of different oxygen content groups on CO_2 capacities.

Table S3. Summary of BET specific surface area and CO_2 capture performance comparison of various solid physical adsorbent.

**Sample**	**Source**	**S_{BET} (m^2 g^{-1})**	**CO_2 (mg g^{-1}) 1 Bar, 273K**	**S_{CO2}/S_{N2} (IAST)**	**Ref.**
ACRF-40c-85-800	porous resorcinol-formaldehyde resin	1944	271	17.6 [a]	This work
PC-2-3	Biomass tar	1829	221	19.7 [a]	[4]
NPC-4-600	triazine-based porous organic polymers	1518	207	34.5 [a]	[5]
FC4	polyimide	941	178	14.2 [b]	[6]
PS-450-2	petroleum coke	1666	261	17 [b]	[7]
NAC-450-1.5	walnut shell	1687	230	24.67 [b]	[8]

[a] Measured at 273 K; [b] Measured at 298 K.

Micropore diffusion model:

$$\frac{q_t}{q_e} = \frac{6}{\sqrt{\pi}}\sqrt{\frac{D_C}{r_c^2}} - \frac{3D_C t}{r_c^2} \qquad \text{Equation S(1)}$$

where D_c is the micropore diffusion parameter (cm^2/s) and r_c is the particle diameter of the adsorbent (s)

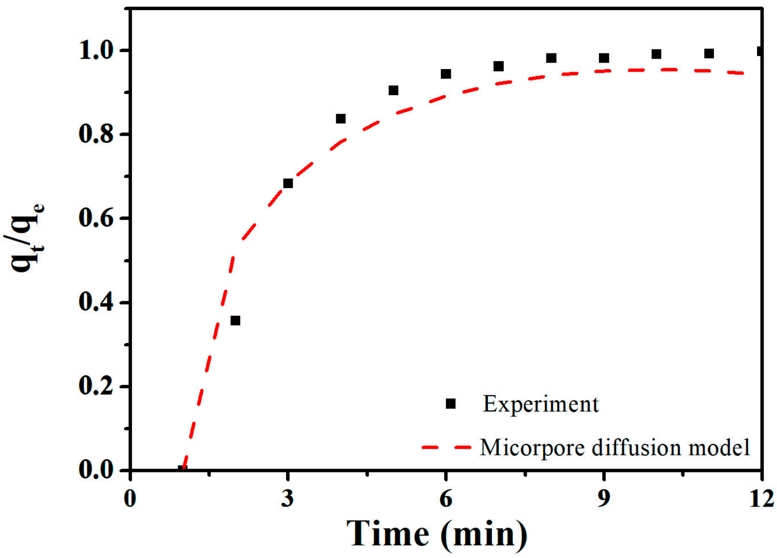

Figure S5. CO_2 adsorption kinetics of ACRF-40C-85%-800 fitted by micropore diffusion model.

Table S4. Parameters of micropore diffusion model for CO_2 adsorption.

Sample	Micropore Diffusion Parameter	
ACRF-40C-85%-800	D_c (cm²/s)	0.238
	r_c (cm)	2.60
	R^2	0.971

Reference:

1. Goel, C.; Bhunia, H.; Bajpai, P.K. Resorcinol–formaldehyde based nanostructured carbons for CO_2 adsorption: Kinetics, isotherm and thermodynamic studies. *RSC Adv.*, **2015**, *5*, 93563–93578.
2. Han, J.; Xu, G.; Dou, H.; MacFarlane, D.R. Porous Nitrogen-Doped Carbon Microspheres Derived from Microporous Polymeric Organic Frameworks for High Performance Electric Double-Layer Capacitors. *Chem. Eur. J.* **2015**, *21*, 2310–2314.
3. Lee, J.-S.M.; Briggs, M.E.; Hasell, T.; Cooper, A.I. Hyperporous carbons from hypercrosslinked polymers. *Adv. Mater.* **2016**, *28*, 9804–9810.
4. Yuan, H.; Chen, J.; Li, D.; Chen, H.; Chen, Y. 5 Ultramicropore-rich renewable porous carbon from biomass tar with excellent adsorption capacity and selectivity for CO_2 capture. *Chem. Eng. J.* **2019**, *373*, 171–178.
5. Shao, L.; Li, Y.; Huang, J.; Liu, Y.N. Synthesis of triazine-based porous organic polymers derived N-enriched porous carbons for CO_2 capture. *Ind. Eng. Chem. Res.* **2018**, *57*, 2856–2865.
6. Chen, C.; Huang, H.; Yu, Y.; Shi, J.; He, C.; Albilali, R.; Pan, H. Template-free synthesis of hierarchical porous carbon with controlled morphology for CO_2 efficient capture. *Chem. Eng. J.* **2018**, *353*, 584–594.
7. Rao, L.; Liu, S.; Chen, J.; Wang, L.; An, L.; Yang, P.; Hu, X. Single-step synthesis of nitrogen-doped porous carbons for CO_2 capture by low-temperature sodium amide activation of petroleum coke. *Energy Fuels* **2018**, *32*, 12787–12794.
8. Yang, Z.; Zhang, G.; Xu, Y.; Zhao, P. One step N-doping and activation of biomass carbon at low temperature through $NaNH_2$: An effective approach to CO_2 adsorbents. *J. CO_2 Util.* **2019**, *33*, 320–329.
